# Hypoxia Transcriptomic Modifications Induced by Proton Irradiation in U87 Glioblastoma Multiforme Cell Line

**DOI:** 10.3390/jpm11040308

**Published:** 2021-04-16

**Authors:** Valentina Bravatà, Walter Tinganelli, Francesco P. Cammarata, Luigi Minafra, Marco Calvaruso, Olga Sokol, Giada Petringa, Giuseppe A.P. Cirrone, Emanuele Scifoni, Giusi I. Forte, Giorgio Russo

**Affiliations:** 1 Institute of Molecular Bioimaging and Physiology–National Research Council (IBFM-CNR), 90015 Cefalù, Italy; valentina.bravata@ibfm.cnr.it (V.B.); marco.calvaruso@ibfm.cnr.it (M.C.); giusi.forte@ibfm.cnr.it (G.I.F.); giorgio.russo@ibfm.cnr.it (G.R.); 2 Laboratori Nazionali del SUD, Istituto Nazionale di Fisica Nucleare (LNS-INFN), 95100 Catania, Italy; petringa@lns.infn.it (G.P.); pablo.cirrone@lns.infn.it (G.A.P.C.); 3 Biophysics Department, GSI Helmholtzzentrum für Schwerionenforschung GmbH, 64291 Darmstadt, Germany; w.tinganelli@gsi.de (W.T.); o.sokol@gsi.de (O.S.); 4 Trento Institute for Fundamental Physics and Applications (TIFPA), Istituto Nazionale Fisica Nucleare (INFN), 38123 Trento, Italy; Emanuele.Scifoni@tifpa.infn.it

**Keywords:** transcriptome, hypoxia, glioblastoma, proton therapy, omic science

## Abstract

In Glioblastoma Multiforme (GBM), hypoxia is associated with radioresistance and poor prognosis. Since standard GBM treatments are not always effective, new strategies are needed to overcome resistance to therapeutic treatments, including radiotherapy (RT). Our study aims to shed light on the biomarker network involved in a hypoxic (0.2% oxygen) GBM cell line that is radioresistant after proton therapy (PT). For cultivating cells in acute hypoxia, GSI’s hypoxic chambers were used. Cells were irradiated in the middle of a spread-out Bragg peak with increasing PT doses to verify the greater radioresistance in hypoxic conditions. Whole-genome cDNA microarray gene expression analyses were performed for samples treated with 2 and 10 Gy to highlight biological processes activated in GBM following PT in the hypoxic condition. We describe cell survival response and significant deregulated pathways responsible for the cell death/survival balance and gene signatures linked to the PT/hypoxia configurations assayed. Highlighting the molecular pathways involved in GBM resistance following hypoxia and ionizing radiation (IR), this work could suggest new molecular targets, allowing the development of targeted drugs to be suggested in association with PT.

## 1. Introduction

Glioblastoma Multiforme (GBM) is the most malignant and the most common tumor among glial neoplasms. It is characterized by an anaplastic, poorly differentiated, and highly cellular grade IV astrocytoma with a peak of incidence between 45 and 70 years [1]. Moreover, GBMs have a poor prognosis, and 5-year survival is less than 10% [2] due to treatment plan failures, often described in GBM patients.

Furthermore, GBM undergoes malignant progression under hypoxic conditions [3]. Hypoxia is a pathophysiological condition that generally arises due to the rapid proliferation of cancer cells as they outgrow their blood supply, therefore depleting cells of nutrients and available oxygen [1,4]. This condition is a feature found in several tumors, and it represents an indication of a poor prognosis. Indeed, hypoxia contributes to give strong radioresistance and chemoresistance, alters the tumor cells’ metabolism, generates strong genome instability, increases angiogenesis and vasculogenesis, and contributes to the formation of the cancer stem cells (CSCs) and circulating tumor cells (CTCs) involved in metastasis formation [1,5]. The hypoxic microenvironment is protective for the tumor and it represents an unfavorable risk element for the radiotherapy’s (RT) clinical outcome, as hypoxic tumors require higher radiation doses to achieve an effective cell killing rate, compared to normoxic ones. The increase in radioresistance in hypoxic tumors, such as GBM, is quantified by the oxygen enhancement ratio (OER), which is the ratio of iso-effective doses in hypoxic and fully oxygenated conditions to produce the same biological effect [1,6,7,8]. As expected, the OER value is strictly dependent on the linear energy transfer (LET) of a specific radiation. Generally, low-LET ionizing radiations, such as photons, elicit tumor cell-killing mainly through indirect effects (e.g., ROS generation), and their efficacy is more susceptible to the tissue’s oxygen concentration. On the contrary, high-LET radiations primarily induce a direct effect on their targets and cell damage is less dependent on oxygen concentration [9]. Thus, tumor hypoxia substantially diminishes the efficacy of conventional anticancer approaches.

The current ASTRO standard guidelines for GBM care are based on surgical resection, conventional RT (60 Gy delivered by 2 Gy daily fractions), and chemotherapy with daily temozolomide (TMZ) administration [9]. However, these approaches are not always curative, and the GBM patient median survival time remains 14.6 months [10]. In this sense, proton therapy (PT) shows better ballistic precision and higher dose conformity than conventional RT, and it could be proposed as a promising treatment modality for GBM cancer [10,11,12]. Further RT strategies for the treatment of GBM account also for the application of carbon ions, which exhibit higher efficacy in terms of radiobiological response than protons. Moreover, encouraging results have been generated using magnetic hyperthermia (MHT) combined with RT to radiosensitize the hypoxic cells of GBM. However, both carbon ions and MHT are still under investigation in several clinical trials [13,14,15]. 

Furthermore, the discovery of new biological biomarkers is needed to perform more successful treatment plans against specific molecular subtypes, and it would be helpful to take into account the GBM genomic features. Then, molecular markers could be considered integral parts of tumor assessment in modern neuro-oncology, helping clinicians to make therapeutic and clinical decisions for GBM patients [2].

Considering these assumptions, this study’s main aim was to analyze the U87 GBM cell line’s response to PT treatment under induced acute hypoxia. Cell survival curves to increasing PT doses under normoxia/acute hypoxia were constructed, to verify the major radioresistance under reduced O_2_ concentrations. In addition, whole-genome gene expression profiling (GEP) analysis was performed on hypoxic samples subjected to RT with a low (2 Gy) and a high (10 Gy) dose. Then, on one hand, the common dose-independent hypoxic response to the PT stress was discussed, whereas, on the other hand, the two 2 Gy and 10 Gy hypoxic samples were compared with respective normoxic treated samples. Thus, the most statistically and biologically relevant deregulated pathways were described for the configurations analyzed and some clinically significant biomarkers were discussed.

## 2. Materials and Methods

### 2.1. GSI Hypoxic Chambers

The GSI hypoxic chambers were produced from single pieces of polyetheretherketone (PEEK). Each chamber had a parallelepiped shape, with one side being an irradiation window of 1 mm thickness (water-equivalent thickness of 1.23 mm). 

The chamber was closed from the top with a transparent polymethylmethacrylate (PMMA) lid. The two chamfers (one in the bottom and one on the top cover) gave the possibility to position the polyvinyl-chloride sample ring with an internal diameter of 24 mm and a thickness of 3 mm. 

For the sample preparation, both of the ring sides were covered with a gas-permeable foil of 25 μm thickness (BioFolie25, In Vitro Systems and Services, Göttingen, Germany). Every layer corresponded to a water-equivalent thickness of 47 µm. 

To reach the desired level of hypoxia, the chambers containing sample rings were sealed, attached to the external bottle with the gas mixture, and flushed for two hours at a rate of approximately 200 mL/min. Samples in normoxia were also irradiated inside the chambers, but without flushing. In this experiment, a mixture of 94.8% N_2_, 0.2% O_2_, and 5% CO_2_ was used for the hypoxic conditions. 

The gas flow was measured at the gas outlet with a mass flow meter calibrated for nitrogen (Vögtlin Instruments AG, Muttenz, Switzerland). Previous studies to determine the required time and gas flow to reach the medium’s planned oxygen state were done using a needle-type housing optical O_2_ microsensor (Pre-Sens, Regensburg, Germany) [16]. 

### 2.2. Cell Culture Preparation and Proton Irradiation Set-Up

Biological samples were prepared 24 h before the irradiation as follows. First, a circle of biofoil was attached to each sample ring using joint grease (Karl Roth, Karlsruhe, Germany), with its hydrophilic side facing the inner part of the ring. U87 GBM cells (European Collection of Authenticated Cell Cultures (ECACC), Public Health England, Porton Down Salisbury, UK) were trypsinized and resuspended inside the growth medium. The cell concentration was adjusted to the value of approx. 33.3 × 10^4^ cells/mL, and 1.5 mL of the resulting cell suspension was transferred inside the ring. At the last step, each ring was closed with another circle of biofoil, transferred into a Petri dish, and incubated until the irradiation day. 

Two hours before irradiation, rings were placed inside the chambers and gassed as described in the previous section. Irradiation of U87 cell line under normoxia conditions was performed as previously reported [11].

Samples were irradiated at CATANA proton therapy facility of INFN-LNS in the middle of a 62 MeV proton spread-out Bragg peak with increasing PT doses (1–10 Gy) [17]. In particular, samples in normoxia were irradiated with doses of 1, 2, 3, 4, and 6 Gy, while the hypoxic samples received doses of 2, 4, 6, and 10 Gy.

### 2.3. Clonogenic Assay

Following irradiation, cells were trypsinized, counted, and re-seeded into 6-well plates in triplicate. The number of re-seeded cells was estimated to re-seed 100 living cells accounting for the expected survival. After 10 days of incubation, the colonies were fixed and stained with 0.5% crystal violet dye in 95% methanol in water. The stained colonies were counted manually, and those containing at least 50 cells were considered as surviving. 

### 2.4. Whole-Genome cDNA Microarray Expression Analysis

To study the biological processes activated in U87 GBM cell line irradiated in the middle of a spread-out Bragg peak with 2 and 10 Gy doses of proton during acute hypoxia, we performed whole-genome cDNA microarray gene expression analyses as previously described [18], comparing samples of interest to hypoxic samples not exposed to RT. Twenty-four hours after PT, U87 GBM cells were harvested, counted, and the pellet stored immediately at −80 °C. Total RNA was extracted from cells using Trizol and the RNeasy mini kit (Invitrogen). RNA concentration and purity were determined spectrophotometrically using a Nanodrop ND-1000 (Thermo Scientific Open Biosystems, Lafayette, CO, USA) and then labeled and hybridized onto Whole Human Genome 4 × 44 K microarray GeneChips (Agilent Technologies Santa Clara, CA, USA) containing all known genes and transcripts of an entire human genome according to the Agilent Two-Color Microarray-Based Gene Expression Analysis protocol. Microarray images were acquired with a DNA Microarray Scanner with Sure Scan High-Resolution Technology (Agilent Technologies Santa Clara, CA, USA). Background correction and normalization, as well as statistical data analyses of the gene expression profiles (GEPs), were performed using Feature Extraction 9.5 and GeneSpring GX 13.0 software (Agilent Technologies Santa Clara, CA, USA). Genes were identified as being differentially expressed if they showed a fold change (FC) of at least 2 with a *p* value < 0.05 compared with U87 untreated cells used as reference. The data discussed in this publication have been deposited in the National Center for Biotechnology Information Gene Expression Omnibus (GEO) [19] and are accessible through GEO Series accession numbers (GSE162986). Microarray data are available in compliance with Minimum Information About a Microarray Experiment (MIAME) standards. 

Finally, we studied biological pathways regulated by the genes belonging to the differentially expressed gene lists obtained by GEP analyses, firstly using the Database for Annotation, Visualization and Integrated Discovery (DAVID) network building tool (https://david.ncifcrf.gov/tools.jsp (accessed on 16 April 2021)), which provides a comprehensive set of functional annotations for investigators to study the biological content captured by high-throughput technologies such as microarray analyses and secondly by using the PubMatrix tool to confirm our assumptions [20]. Since the list of deregulated pathways was long and complex, we decided to describe only the top 15 significantly upregulated pathways.

## 3. Results

### 3.1. Survival Curves

**Figure 1:**
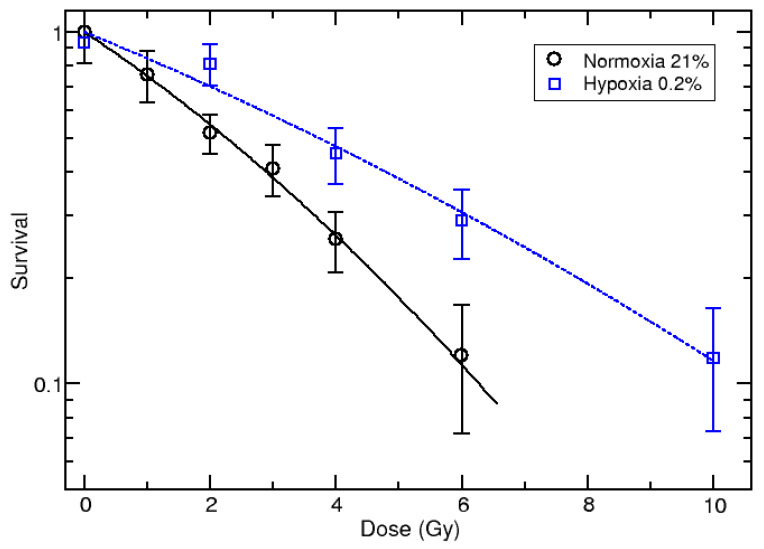
Survival curve of U87 cells irradiated in normoxia (O_2_ = 21%) from updated data [9] and hypoxia (O_2_ = 0.2%) with 62 MeV proton beam. The measured values are shown with black circles (normoxia) and blue squares (hypoxia), and the lines correspond to the linear–quadratic fit of measured data.

Figure 1 shows the survival curve for U87 cell line, irradiated in the middle of a 62 MeV proton spread-out Bragg peak in hypoxia (0.2% O_2_), compared with the normoxia data (21% O_2_). Each point represents the average of two independent repetitions. Both sets of data were fitted using the linear–quadratic approach, describing the survival as a function of dose as lnS = −αD–βD^2^. The plot demonstrates a substantial increase in cell survival in hypoxic conditions with an OER_S=10%_ = 1.69 ± 0.36.

### 3.2. Overview of cDNA Microarray Gene Expression Analyses under PT/Hypoxia Conditions

As described above, we analyzed the GEPs induced by PT irradiation using 2 and 10 Gy doses of IR on the U87 GBM cell line exposed to acute hypoxia by using the GSI hypoxia chambers, able to reproduce hypoxia in vitro with the following conditions: 94.8% N_2_, 5%CO_2_, 0.2% O_2_. 

The decision to consider these two doses, one low and one high, was related to the fact that 2 Gy is the daily dose delivered during fractionated RT treatments, while 10 Gy represents a dose of clinical interest for comparisons with other GEP analyses performed by our research group and also according to the hypofractionated stereotactic radiotherapy (hSRT) regimens that were recently performed [11,21,22].

In detail, we analyzed the following configurations: (**i**) U87 cell treated with 2 Gy under acute hypoxia (hereafter named U87_2Gy_Hyp); (**ii**) U87 cell treated with 10 Gy under acute hypoxia (named U87_10Gy_Hyp). 

Comparative differential gene expression analyses revealed that a conspicuous number of genes had significantly altered expression levels by two-fold or greater, compared to the hypoxic non-irradiated samples, as displayed in Table 1. 

On the other hand, considering that the number of genes selected with a more stringent statistical significance (fold change > 5) was too small to carry out an exhaustive network analysis, only GEPs with an f.c. > 2 were analyzed and described. However, selected genes, with high fold change values, are also described in the Discussion section to highlight their interesting roles in cell responses to PT under acute hypoxia.

### 3.3. Pathway Analysis of GEP Lists under Combined PT/Hypoxia Conditions

Up- and downregulated transcripts for each configuration analyzed in this study were selected and grouped according to their involvement in specific biological pathways using the DAVID tool, as previously reported [23]. Since the list of deregulated pathways was long and complex, we decided to describe only the top 15 significantly upregulated pathways after 2 and 10 Gy doses of proton to select specific biomarkers strictly linked to the treatments (Table 2 and Table 3). 

In particular, as shown in Table 2, after 2 Gy of PT, the U87 hypoxic cells were able to deregulate a set of genes, mainly involved in pro-survival cellular signals and cancer development (Table 2). In summary, some of the genes included in the GEP lists control the cell fate (i.e., cell cycle and p53 signaling pathway); others are related to tumor progression, cell–cell communication, angiogenesis, invasiveness (i.e., pathways in cancer, VEGF signaling pathway, proteoglycans in cancer, Ras signaling pathway, signaling pathways regulating pluripotency of stem cells, Wnt signaling pathway, focal adhesion); some others participate in multiple intracellular signaling processes associated with different cell activities (i.e., phosphatidylinositol signaling system, inositol phosphate metabolism, and PI3K-Akt signaling pathway). In addition, the Hippo signaling pathway, FoxO signaling pathway, and Rap1 signaling pathway are redundant, as they were found to be related to PT cell response in other studies by our group, and, in our opinion, they need further investigation [11].

Similarly, as reported in Table 3, the U87 GBM hypoxic cells, exposed to 10 Gy of PT, also upregulated 10 common pathways out of the 15 above described. This list represents the dose-independent response of hypoxic cells to PT irradiation (Hippo signaling pathway; proteoglycans in cancer; FoxO signaling pathway; P53 signaling pathway; focal adhesion; PI3K-Akt signaling pathway; signaling pathways regulating pluripotency of stem cells; Wnt signaling pathway; Rap1 signaling pathway and cell cycle; cell cycle).

Furthermore, the other five upregulated pathways represent the high dose (10 Gy)-related response to irradiation. These specific signatures seem to be involved in the post-irradiation damage control, as some pathways are related to the immunological balance, cell communication, and bystander effect (tumor necrosis factor, TNF, mTOR signaling, endocytosis) [24,25,26,27], whereas the AMPK signaling pathway has been recognized to mediate stress responses to facilitate autophagy [28] and the neurotrophin pathway’s upregulation could be involved in neurogenesis and/or neurorepair processes, induced by both radiation and hypoxia exposure [29].

**Figure 2:**
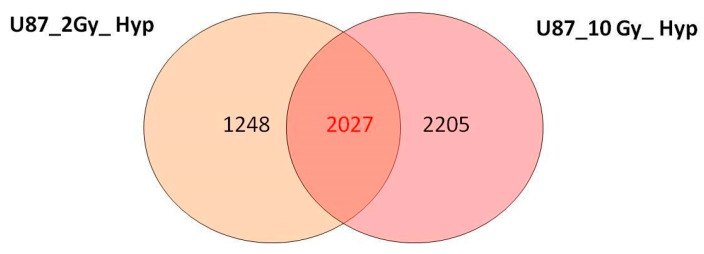
Venn diagrams showing the number of unique and shared differentially expressed genes (DEGs) after exposure to 2 and 10 Gy of PT under acute hypoxia.

To specifically highlight differentially expressed, shared genes between the U87 GBM cells irradiated with 2 and 10 Gy doses of proton under acute hypoxia, we constructed Venn diagrams, as shown in Figure 2, directly using the starting gene lists by two-fold.

As shown, many genes were commonly deregulated in the two configurations assayed, hereafter named 2027-gene signature, linked to the proton cell response under hypoxia. 

Table 4 reports the result of DAVID analysis performed on the 2027-gene signature of commonly deregulated genes. This list of the top 10 significant pathways represents the dose-independent response of hypoxic cells to PT, and, with two exceptions (endocytosis and VEGF signaling pathway), shows the pathways already found in the 2 and 10 Gy pathway list comparison (Table 2 and Table 3). 

### 3.4. Commonly Deregulated Genes and Pathways among PT-Treated Samples under Normoxia vs. Hypoxia Condition

**Figure 3:**
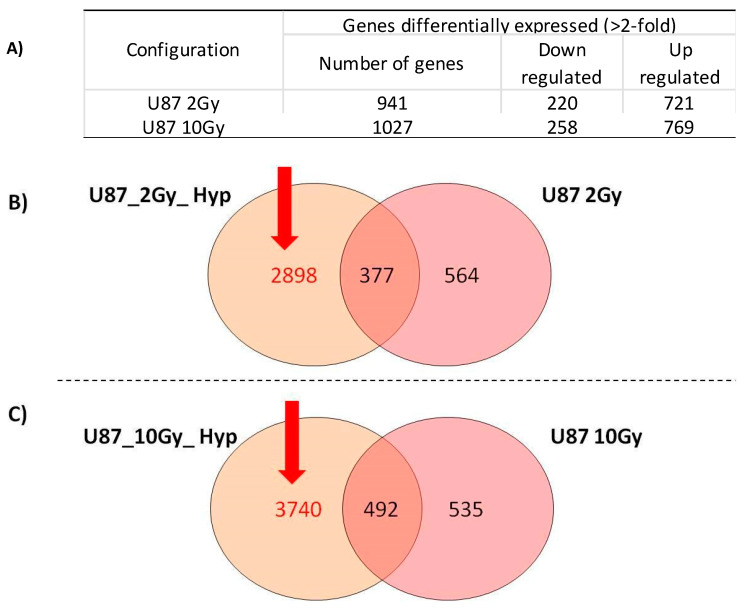
(**A**) Number of genes significantly deregulated by 2-fold in U87 GBM cells exposed to 2 and 10 Gy of PT under normoxia condition. (**B**) Venn diagrams showing the number of unique and shared differentially expressed genes (DEGs) after exposure to 2 Gy of PT under acute hypoxia or normoxia. (**C**) Venn diagrams showing the number of unique and shared differentially expressed genes (DEGs) after exposure to 10 Gy of PT under acute hypoxia or normoxia. In red, the two gene signatures (2898- and 3740) linked to the hypoxia condition are highlighted.

As previously reported, we already analyzed the gene expression changes in the U87 GBM cell line, induced by PT with the doses of 2 and 10 Gy, under normoxia conditions (Figure 3A) [9]. Then, here, we also compared GEPs from PT-treated samples with 2 and 10 Gy under normoxia vs. hypoxia conditions. As shown in Figure 3B,C, some genes were commonly deregulated by hypoxia and normoxia, being O_2_-independent, under the same doses provided (377- and 492-gene signatures).

To study gene lists strictly related to acute hypoxia conditions in the U87 cells, we analyzed the following two 2898- and 3740-gene signatures, by using the DAVID tool (Figure 3B,C). The top ten molecular upregulated pathways were selected and then analyzed using the Pubmatrix tool as previously described [23]. In this way, bibliographic relationships between the selected pathways and some selected queries, such as hypoxia, GBM, RT, proton therapy, cancer, ionizing radiation, cell death, cell cycle, and Hif1-alpha, were analyzed. The resulting data, useful to test our assumptions, are reported in Table 5 and Table 6.

As shown, some pathways were shared between the two 2898- and 3740-gene signatures (platelet activation, Wnt signaling pathway, Ras signaling, proteoglycans in cancer, and protein processing in the endoplasmic reticulum); thus, these are dose-independent signals activated by hypoxia. Otherwise, the other cellular signaling processes are the 2 Gy dose-dependent pathways upregulated under acute hypoxia: ubiquitin-mediated proteolysis, regulation of actin cytoskeleton, Rap1 signaling pathway, glutamatergic synapse, and FoxO signaling pathways (Table 5). The 10 Gy dose-dependent pathways upregulated under acute hypoxia are as follows: PI3K-Akt signaling pathway, insulin signaling pathway, endocytosis, focal adhesion, and cAMP signaling pathway (Table 6). 

## 4. Discussion

Literature data report that GBM care failures occur due to the resistant responses to multiple treatment approaches (such as chemo- and RT). Thus, new strategies to overcome resistance to treatment are needed in the care of GBM patients. In particular, GBM aggressiveness is often related to extensive hypoxic regions, hallmarks of these tumors that certainly contribute to their highly malignant phenotype, seriously affecting the patient’s prognosis. Tumor cells are resistant to chemo-/radiotherapy and are protected by hypoxia due to disordered and incomplete vascularization. The abnormal and malfunctioning vessels play a critical role in generating necrotic and hypoxic regions, where residing cancer stem cells are protected from therapeutic agents, facilitating tumor aggressiveness as well as GBM stem cell proliferation [30].

With all these assumptions, the primary aim of this work was to analyze the GBM U87 cell line’s molecular response to PT treatment, under acute hypoxia (0.2% O_2_).

Figure 1 shows the U87 survival curves under normoxia (21% O_2_) and acute hypoxia conditions (0.2% O_2_), confirming the consistent gain in radioresistance expected under oxygen deprivation, with an OER_S=10%_ = 1.69 ± 0.36 [11].

Then, we performed a transcriptomic study, i.e., GEP, by using the whole-genome cDNA microarray methodology. In particular, the GEP lists obtained in response to combined hypoxia/2 Gy PT and hypoxia/10 Gy PT were analyzed and genes were grouped according to their involvement in specific biological pathways. Consequently, the top 15 upregulated pathways were selected to search for specific biomarkers, strictly deregulated after treatment (Table 2 and Table 3).

As shown in Table 2 and Table 3, a large number of the selected pathways (10 out of the 15) were commonly upregulated after both 2 and 10 Gy doses of proton under the acute hypoxia condition: Hippo signaling pathway, proteoglycans in cancer, FoxO signaling pathway, P53 signaling pathway, focal adhesion, PI3K-Akt signaling pathway, signaling pathways regulating the pluripotency of stem cells, Wnt signaling pathway, Rap1 signaling pathway, and cell cycle. However, to better describe the hypoxic U87 dose-independent signature in response to PT treatment, we also constructed Venn diagrams using the starting deregulated gene lists (U87_2Gy_Hyp and U87_10Gy_Hyp) by two-fold.

As shown, many genes were commonly deregulated in the two configurations assayed, hereafter named the 2027-gene signature, and Table 4 reports the respective top 10 significant pathways, as identified by DAVID analysis. With two exceptions (PI3K-Akt signaling pathway and cell cycle), this list confirms the involvement of the other 8 out of 10 above-mentioned pathways, which emerged from the comparison of Table 2 and Table 3, to which endocytosis and the VEGF signaling pathway were added. Thus, these pathways represent the dose-independent molecular response of the hypoxic U87 cells to PT stress, using both a low and a high dose. Overall, this signature is rich in pro-survival signals, able to regulate cell fate, progression, and invasiveness. These data are in line with the considerable aggressiveness often described for U87 GBM cells. 

Some of these pathways are known to be related to the IR response in several cancer subtypes (p53 signaling pathway, focal adhesion, Wnt signaling pathway). On the other hand, among these ten pathways, some others were recently associated with the RT and PT response (Hippo signaling pathway, proteoglycans in cancer, FoxO signaling, signaling pathways regulating the pluripotency of stem cells, and Rap1 signaling pathway), as was also reported by our group in previous experiments [11,31,32]. Their roles in the response to hypoxia and radiation stress are described. As is known, Wnt and Ras signaling regulate proliferation, motility, and survival in a variety of cancers and several literature data report their activation after hypoxia as well as after radiation exposure [33,34,35,36].

Little bibliographic information is available about the Rap1 role. It encodes a protein involved in a complex regulating telomere length, possibly involved in the activation of the senescence process, often induced by IR [37]. More specifically, RAP1 contributes to maintaining genome stability by protecting telomeric DNA ends from non-homologous end joining and from homologous recombination, which can alter telomere length [38]. Rap1 level is described as affected by cellular aging and oxidative stress in cancers, including GBM, and it was also shown by our group to be related to the cell radiation response. Moreover, Sayyah et al. demonstrated a critical role of Rap1A in in vivo GBM tumor growth, as the induced integrin activation and the downstream cell signaling were described as crucial factors in GBM cell proliferation [39].

In addition, as we also recently described, Hippo signaling is actively involved in the cell response to PT [10]. Its dysregulation represents a common event in many cancers, including glioma [40], and its transcriptional coactivators, the YAP and TAZ proteins, were implicated as drivers of GBM progression and then suggested as therapeutic targets by Liu et al. [41]. Indeed, their hyperactivation is associated with resistance to conventional chemotherapies, radiotherapy, and targeted therapies [31]. In this sense, it was highlighted that TAZ inhibition favors radiation-induced senescence, increasing the GBM RT effectiveness [42].

On the other hand, proteoglycans are often described as able to drive cell–cell communication and cell–microenvironment interactions. They are abundant in the brain and have known roles in normal neurological development, as they can regulate the proliferation and maintenance of neural progenitor cells, also through Wnt signaling. However, changes in proteoglycan core proteins, often described in many cancers, including GBM, are associated with the acquisition of a mesenchymal tumor trait [43]. In addition, they could interfere with angiogenesis and autophagy signaling pathways; thus, considering that, under hypoxia and starvation conditions, tumors use angiogenesis to provide nutrients, this pathway could have an interesting role in tumor survival under hypoxia [44,45], as also supported by the VEGF pathway’s involvement in this signature [28].

Regarding the radiobiological role of FoxO signaling, fewer data are available. However, the Fox proteins are TFs inhibited by the PI3K/Akt/mTOR pathway and share many target genes with the p53 protein. Interestingly, FoxO proteins or loss of functional p53 maintains the stemness of GBM stem cells and survival after IR treatment [46]. It has been related to modulating hypoxia-induced autophagy [47], whereas FoxG1 overexpression restored the cell viability after TMZ treatment, as described by Wang et al. [48]. 

Moreover, regarding stem cell signaling, it is well known the role of GBM subpopulation stem-like cells (GSCs) with self-renewal properties, involved in recurrence and in conferring resistance to therapeutic interventions producing DNA damage in GBM (i.e., RT), through the constitutive upregulation of the DNA damage response (DDR) pathway [49,50,51]. 

The role of focal adhesion is reported to be related to the epithelial-to-mesenchymal transition (EMT), migration, and invasion [52], whereas, regarding the endocytosis pathway, the modification of endocytosis fluxes is described in response to stresses, including hypoxia and IR, as adaptation and communication with the surrounding microenvironment, using nutrients and molecules [53]. 

Thus, as the above-described molecular signature is responsible for conferring the radioresistance gain observed in Figure 1; it is of interest for the possibility of developing new targeted molecules to be suggested for combined treatment with PT to overcome hypoxia-related tumor radioresistance. 

In addition, in the second part of this work, we also compared the GEP lists obtained under combined hypoxia/PT treatment with 2 or 10 Gy vs. the previously obtained GEP lists obtained, treating the U87 cells with 2 or 10 Gy under normoxia conditions [11]. As shown in Figure 3B,C, for each delivered dose, some DEGs were commonly deregulated, being O_2_-independent (377- and 492-gene signatures); some others were normoxia-related DEGs (564- and 535-gene signatures), whereas a larger number of genes were altered by the acute hypoxia condition, consisting of the two 2898- and 3740-gene signatures. 

Once again, we analyzed the last two DEG lists, which instead describe the dose-dependent U87 response to 2 Gy or 10 Gy under acute hypoxia. The Pubmatrix analysis identified the top 10 statistically relevant pathways (selected by the DAVID tool), involved in hypoxia and other interesting conditions related to radiobiology (hypoxia, RT, PT, IR, cell death, cell cycle, Hif1-alpha) (Table 5 and Table 6). The data collected confirm the knowledge presented in the literature about their activation after oxygen deprivation [53,54]. 

**Figure 4:**
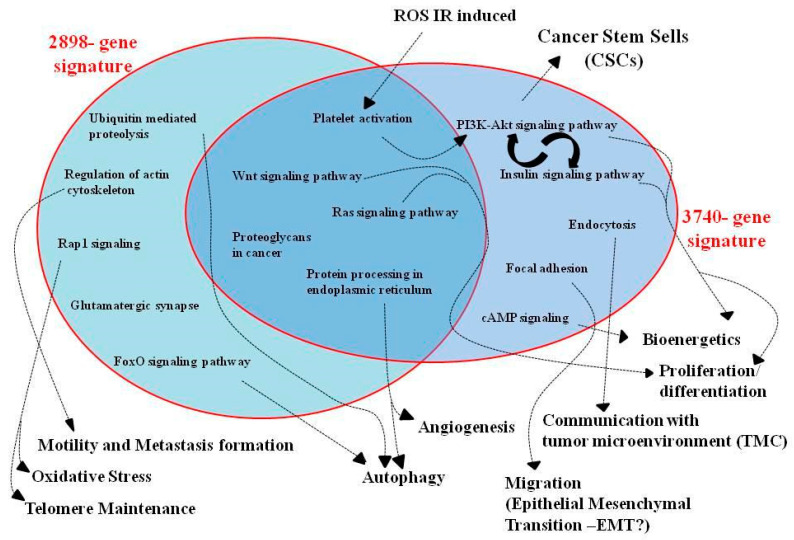
The figure summarizes the main intracellular networks of the 2898- and 3740-gene signatures highlighted in this work as activated in U87 GBM cells after hypoxia and PT exposure.

Figure 4 further presents shared and unique pathways deriving from the 2898- and 3740-gene signatures.

Again, common pathways between 2 Gy and 10 Gy response were as follows: platelet activation, Wnt signaling pathway, Ras signaling, proteoglycans in cancer, and protein processing in the endoplasmic reticulum. In particular, among these, we did not describe the role of platelet-derived growth factor receptor, which acts in combination with phosphatidylinositol 3-Kinase (PI3K) and AKT pathways in response to hypoxia, induced by an ischemic event, which produces O_2_ deprivation and reactive oxygen species (ROS), which are also generated by radiation exposure [53,54]. 

On the other hand, less information is available regarding the “protein processing in the endoplasmic reticulum” pathway, although the endoplasmic reticulum stress is described in the literature as induced by TMZ in GBM. Indeed, Lee et al. reported that hyperoxia resensitizes TMZ-resistant GBM cells to TMZ, by abrogating the hypoxia-induced, unfolded protein response related to protective mechanisms [55]. However, its role needs to be better evaluated. 

As shown in Table 5 and Figure 4, other cellular signaling processes were regulated under acute hypoxia in the 2898-gene signature in response to a dose of 2 Gy: ubiquitin-mediated proteolysis, regulation of actin cytoskeleton, Rap1 signaling pathway, glutamatergic synapse, and FoxO signaling pathways. 

The ubiquitin-mediated proteolysis could be related to autophagy activation, a process often involved in tumor response to radiation [32], although also HIF-1α promotes autophagic proteolysis of the Dicer complex and enhances tumor metastasis in GBM cells [56]. 

Moreover, hypoxia controls cytoskeletal dynamics to promote local invasion through actin cytoskeleton remodeling, as reported by Fujimura and colleagues, who identified Cyclin G2 (upregulated in 2898-gene signature with a high fold change value of 5.49), as a driver gene in promoting local invasion, by cytoskeletal remodeling under hypoxia in GBM [57]. Interestingly, Lee and colleagues also reported its role in promoting cell adhesion and actin cytoskeletal polarization, affecting cell migration and metastasis formation [58]. No relevant information regarding glutamatergic synapse modulation and hypoxia or radiation cell response is available in the literature, thus representing a new, interesting issue in radiobiological investigations. 

Table 6 displays the upregulated pathways by the combined treatment with 10 Gy of PT and acute hypoxia, deriving from the 3740-gene signature: PI3K-Akt signaling pathway, insulin signaling pathway, endocytosis, focal adhesion, and cAMP signaling pathway (Table 6). Their role has already been discussed above, except for the insulin signaling pathway, for which a close connection with the PI3K-Akt signaling pathway can be found, in regulating numerous intracellular pro-survival and tumor progression processes. In particular, a biomarker of this network is certainly the glycogen synthase kinase-3 (GSK-3), regulated by the insulin signaling pathway. In GBM, under hypoxic conditions, the PI3K/Akt pathway regulates glucose metabolism [59], conditioning tumor growth, angiogenesis, and invasion [60]. 

Regarding the cAMP signaling pathway, Chhipa and colleagues recently reported that it could promote GBM bioenergetics and tumor growth [61], whereas our group has already described it as deregulated after RT [11,62]. Indeed, IR is known to activate the transcription of genes, through the presence of cAMP-responsive elements (CREs) in their promoters, to guide cell fate and survival after radiation exposure [62,63,64]. Finally, it was reported that cancer-associated stress, including hypoxia, chronically activates the bioenergetic sensor AMP kinase (AMPK), as a pro-survival signal [65,66].

## 5. Conclusions

As described above, the main aim of this work was to shed light on cell signaling networks involved during acute hypoxia (0.2% O_2_) in the U87 GBM cell line after PT. The U87 survival hypoxic and normoxic curves demonstrate a radioresistance gain, quantified with an OER_S=10%_ = 1.69 ± 0.36. Then, our group elucidated the molecular response under the hypoxia condition. 

In summary, our study reveals the activation of intracellular networks which, overall, are able to regulate pro-survival cell fate, progression, and invasiveness. These signals are induced by PT itself rather than the specific dose delivered. This work contributes to understanding the radioresistance mechanisms activated by GBM, giving insights into their use for developing targeted molecules to be suggested in combination with PT to improve the RT success rate. 

## Figures and Tables

**Table 1:** Number of genes significantly deregulated by 2-fold or 5-fold in all the configurations assayed in this work.

**Genes Differentially Expressed (>2-fold)**			
**Configuration**	**Number of Genes**	**Downregulated**	**Upregulated**
U87_2Gy_ Hyp	3275	773	2502
U87_10Gy_ Hyp	4232	1605	2627
**Genes Differentially Expressed (>5-fold)**			
**Configuration**	**Number of Genes**	**Downregulated**	**Upregulated**
U87_2Gy_ Hyp	207	1	206
U87_10Gy_ Hyp	293	119	174

**Table 2:** Top 15 statistically relevant pathways activated in U87 glioblastoma cells exposed to 2Gy PT irradiation under hypoxia condition.

Pathways	Gene Count	*p* Value	Genes
Proteoglycans in cancer	47	9.80 × 10^8^	CAV2, FGFR1, LUM, PPP1R12C, SDC4, MMP2, PDCD4, ITGB1, IQGAP1, PXN, TGFB2, CTNNB1, PTK2, KRAS, ANK2, GAB1, PPP1R12A, PRKACB, THBS1, WNT6, PIK3R1, AKT2, FN1, TWIST1, PIK3R2, ACTB, ROCK1, ROCK2, MAP2K2, MET, ITGA2, ARHGEF12, PPP1CC, FLNC, PPP1CB, STAT3, ITPR1, FLNA, PRKCB, FZD6, PTPN11, CCND1, CBLB, MAPK12, ITGA5, VEGFA, HBEGF
Pathways in cancer	74	5.22 × 10^9^	GNA13, FGF5, FGF7, PTGS2, PGF, STAT5B, NFKB2, MMP2, TGFB2, CTNNB1, EDNRA, CUL2, CASP8, RALB, RARB, PRKACB, WNT6, AKT2, CTBP1, BCR, ROCK1, PTGER4, ROCK2, FADD, RB1, ARHGEF12, DAPK3, CDK2, CTNNA2, PRKCB, CCND1, EP300, GNB2, GNAQ, GNB1, LPAR6, VEGFA, FGFR1, XIAP, GNAI1, PML, BCL2L1, ITGB1, TPM3, PTK2, KRAS, RUNX1, AXIN2, PIK3R1, FN1, APC, PIK3R2, CEBPA, DVL3, EPAS1, MAP2K2, MET, SMAD4, ITGA2, STAT3, COL4A6, DVL1, FZD6, CBLB, CDKN1B, ADCY9, ITGA6, ETS1, BAX, RASSF1, GSK3B, JAK1, ABL1, CRK
Hippo signaling pathway	37	6.31 × 10^9^	YWHAZ, SOX2, BMPR2, LATS1, CTNNB1, TGFB2, DLG4, LIMD1, YAP1, AXIN2, WNT6, APC, ACTB, DVL3, PARD6B, NF2, SMAD4, PPP1CC, SNAI2, YWHAE, PPP1CB, TP73, CTNNA2, FZD6, DVL1, CCND1, YWHAG, YWHAH, CCND3, ID2, CSNK1E, CCND2, BBC3, GSK3B, RASSF1, PARD6G, BMP8B
Focal adhesion	43	6.55 × 10^9^	CAV2, TLN1, XIAP, PGF, PPP1R12C, ARHGAP35, ITGB1, PXN, CTNNB1, MYL9, PTK2, PAK2, COL6A3, PPP1R12A, COL6A2, COL6A1, SHC1, THBS1, RAPGEF1, PIK3R1, PIK3R2, FN1, AKT2, ACTB, ROCK1, ROCK2, MET, ITGA2, FLNC, PPP1CC, PPP1CB, FLNA, COL4A6, PRKCB, CCND1, CCND3, ITGA6, ITGA5, CCND2, ITGA8, GSK3B, VEGFA, CRK
Signaling pathways regulating pluripotency of stem cells	32	1.24 × 10^11^	BMI1, FGFR1, FGFR4, ONECUT1, IL6ST, SOX2, BMPR2, REST, CTNNB1, ACVR1C, PCGF5, KRAS, SKIL, AXIN2, WNT6, PIK3R1, PIK3R2, APC, AKT2, DVL3, MAP2K2, SMAD4, LIFR, STAT3, FZD6, DVL1, ID2, RIF1, MAPK12, GSK3B, JAK1, KAT6A
FoxO signaling pathway	29	7.03 × 10^11^	STK11, PRKAG2, BNIP3, CCNG2, TGFB2, KRAS, PRKAA2, INSR, PIK3R1, PIK3R2, AKT2, IRS2, SGK2, MAP2K2, SMAD4, GRM1, IRS1, CDK2, STAT3, SOD2, CCND1, PLK4, CDKN1B, EP300, MAPK12, CSNK1E, CCND2, SETD7, GADD45B
p53 signaling pathway	18	8.34 × 10^11^	ZMAT3, RRM2B, CCNG1, CCNG2, SESN1, CDK2, TP73, CCND1, CCND3, BBC3, CCND2, BAX, RRM2, CASP8, SIAH1, MDM4, THBS1, GADD45B
Rap1 signaling pathway	39	0.001	FGFR1, FGF5, TLN1, FGFR4, FGF7, GNAI1, PGF, EFNA3, CTNND1, ITGB1, CTNNB1, PFN2, KRAS, RALB, RAPGEF4, RAPGEF2, THBS1, RAPGEF1, INSR, PIK3R1, PIK3R2, AKT2, ACTB, PARD6B, GNAO1, MAP2K2, MET, GRIN2A, SIPA1L3, PRKCB, DOCK4, ADCY9, MAPK12, GNAQ, KRIT1, VEGFA, PARD6G, CRK, CALM1
Cell cycle	25	0.004	FZR1, YWHAZ, E2F4, E2F5, CDC14B, SMAD4, PRKDC, RB1, YWHAE, WEE1, CDK2, TGFB2, CCND1, YWHAG, RAD21, YWHAH, EP300, CDKN1B, CCND3, CCND2, GSK3B, ANAPC7, ABL1, GADD45B, STAG2
Phosphatidylinositol signaling system	21	0.005	IMPAD1, IMPA1, PIK3C2A, SYNJ1, PI4K2B, PIP5K1A, ITPR1, PRKCB, DGKA, MTM1, MTMR14, PIKFYVE, PLCD3, INPP5E, PIP4K2A, MTMR6, IPMK, PIK3R1, INPP5A, CALM1, PIK3R2
Ras signaling pathway	39	0.006	FGFR1, FGF5, FGFR4, FGF7, PGF, EFNA3, ARF6, BCL2L1, KRAS, REL, PAK2, GAB1, RALB, SHC1, PRKACB, INSR, PIK3R1, RASA2, PIK3R2, AKT2, PLA2G16, MAP2K2, NF1, MET, GRIN2A, PRKCB, PTPN11, PLA2G4A, KSR2, GNB2, GNB1, ETS1, ETS2, RASSF1, VEGFA, RAB5A, PLA2G2A, ABL1, CALM1
Wnt signaling pathway	26	0.009	PPP3R1, CTNNB1, CSNK2A1, PRKACB, WNT6, NFATC2, AXIN2, FOSL1, APC, CSNK1A1, TBL1XR1, DVL3, CTBP1, ROCK2, SMAD4, FZD6, DVL1, PRKCB, CCND1, EP300, CCND3, CSNK1E, CCND2, SFRP2, GSK3B, SIAH1
Inositol phosphate metabolism	16	0.01	MINPP1, IMPAD1, IMPA1, PIK3C2A, SYNJ1, PI4K2B, PIP5K1A, MTM1, MTMR14, PIKFYVE, PLCD3, INPP5E, PIP4K2A, MTMR6, IPMK, INPP5A
PI3K-Akt signaling pathway	53	0.01	FGF5, FGF7, PGF, PPP2R5A, EFNA3, PKN3, INSR, GHR, AKT2, SGK2, PKN2, IRS1, CDK2, IFNAR2, CCND1, CCND3, GNB2, LPAR6, CCND2, GNB1, VEGFA, FGFR1, YWHAZ, FGFR4, STK11, BCL2L1, ITGB1, ATF2, PTK2, KRAS, COL6A3, COL6A2, COL6A1, PRKAA2, THBS1, PIK3R1, FN1, PIK3R2, MAP2K2, CREB1, MET, ITGA2, YWHAE, COL4A6, YWHAG, YWHAH, CDKN1B, EIF4E, ITGA6, ITGA5, ITGA8, GSK3B, JAK1
VEGF signaling pathway	14	0.01	PTGS2, MAP2K2, PPP3R1, PXN, PRKCB, PLA2G4A, PTK2, KRAS, MAPK12, VEGFA, NFATC2, PIK3R1, AKT2, PIK3R2

**Table 3:** Top 15 statistically relevant pathways activated in the U87 glioblastoma cells exposed to 10 Gy PT irradiation under acute hypoxia condition.

Pathways	Gene Count	*p* Values	Genes
Endocytosis	53	1.43 × 10^11^	HRAS, CHMP3, RAB5B, CAPZA2, EPS15L1, PIP5K1A, MVB12A, PIP5KL1, VPS4B, DNAJC6, AGAP3, PLD1, HLA-A, HLA-C, HLA-B, HLA-E, LDLRAP1, ACAP3, ACAP2, RAB5A, MDM2, PDCD6IP, SNX12, VPS26B, SH3GL1, CAV2, WASH1, STAM2, ASAP2, PML, ASAP1, HSPA1A, CYTH2, ARF6, CHMP2B, SH3GLB2, RAB11B, RAB11A, NEDD4L, HSPA8, EHD4, GIT1, PARD6B, RAB8A, VTA1, EPS15, AP2A2, AP2A1, HGS, SMURF2, PARD6G, ARAP2, DNM2
Hippo signaling pathway	37	3.38 × 10^10^	YWHAZ, APC2, SOX2, BMPR2, LATS1, CTNNB1, TGFB2, CTGF, DLG4, YAP1, WNT6, PPP2R2C, APC, ACTB, DVL3, PARD6B, NF2, SMAD4, WWTR1, PPP1CC, PPP1CB, TP73, STK3, CTNNA2, DVL1, AMH, CCND1, YWHAH, CCND3, CSNK1E, CCND2, BBC3, RASSF1, PARD6G, BMP8B, BMPR1A, PPP2R2A
Proteoglycans in cancer	45	3.99 × 10^10^	CAV2, FGFR1, HRAS, GRB2, LUM, PPP1R12C, ELK1, RPS6KB2, SDC4, MMP2, PDCD4, IQGAP1, PXN, TGFB2, CTNNB1, CTTN, KRAS, ANK2, GAB1, PPP1R12A, PRKACB, WNT6, PIK3R1, AKT2, TWIST1, PIK3R2, ACTB, ROCK2, MAP2K2, MET, ITGA2, ARHGEF12, PPP1CC, FLNC, PPP1CB, STAT3, FLNA, EIF4B, CCND1, CDKN1A, SDC1, MAPK12, ARAF, HBEGF, MDM2
FoxO signaling pathway	33	8.84 × 10^10^	HRAS, GRB2, STK11, PRKAG2, CCNG2, TGFB2, PRMT1, KRAS, PRKAA2, INSR, PIK3R1, PIK3R2, AKT2, SGK1, MAP2K2, SMAD4, PCK2, IRS1, CDK2, STAT3, SOD2, CCND1, CDKN1A, PLK4, CDKN1B, PLK2, MAPK12, CSNK1E, CCND2, ARAF, MDM2, GADD45B, GADD45A
AMPK signaling pathway	31	9.67 × 10^10^	CAB39L, PFKFB3, STK11, PPP2R5A, LEPR, PPP2R5D, PRKAG2, RPS6KB2, CAMKK1, AKT1S1, FASN, RAB11B, PRKAA2, INSR, PPP2R2C, PIK3R1, PIK3R2, AKT2, RAB2A, RAB8A, PFKL, CREB3, SCD, ADIPOR1, CREB5, EEF2, ACACB, PCK2, IRS1, CCND1, PPP2R2A
p53 signaling pathway	18	0.001	ZMAT3, RRM2B, CCNG2, SESN1, CDK2, TP73, CCND1, CDKN1A, CCND3, BBC3, CCND2, RRM2, BAX, CASP8, MDM2, SIAH1, GADD45B, GADD45A
Focal adhesion	39	0.004	CAV2, TLN1, HRAS, GRB2, PGF, PPP1R12C, ELK1, ARHGAP35, PXN, CTNNB1, MYL9, BCL2, PPP1R12A, COL6A2, SHC1, RAPGEF1, PIK3R1, PIK3R2, AKT2, ACTB, TNXB, ROCK2, MET, ITGA2, BAD, FLNC, PPP1CC, PPP1CB, FLNA, COL4A6, COL4A5, VEGFB, CCND1, LAMA3, CCND3, ITGA6, CCND2, LAMC2, CRK
PI3K-Akt signaling pathway	59	0.004	HRAS, PGF, FGF14, EFNA1, PPP2R5A, PPP2R5D, EFNA3, RPS6KB2, PKN3, MLST8, GNG3, INSR, AKT2, SGK1, PKN2, PKN1, IRS1, CDK2, VEGFB, IFNAR2, CCND1, GNB2, CCND3, CCND2, MDM2, LAMC2, PPP2R2A, FGFR1, YWHAZ, GRB2, STK11, BCL2L1, CDC37, KRAS, BCL2, COL6A2, PRKAA2, PPP2R2C, PIK3R1, PIK3R2, TNXB, CREB3, MAP2K2, MET, ITGA2, NR4A1, CREB5, BAD, PCK2, COL4A6, COL4A5, EIF4B, CDKN1A, ATF4, LAMA3, YWHAH, CDKN1B, EIF4E, ITGA6
Neurotrophin signaling pathway	24	0.01	IRAK1, HRAS, MAP2K2, GRB2, NFKBIB, BAD, IRS1, TP73, ATF4, KRAS, PSEN1, MAPK12, BCL2, BAX, GAB1, PSEN2, SHC1, SH2B1, RAPGEF1, CRK, ARHGDIA, PIK3R1, AKT2, PIK3R2
Signaling pathways regulating pluripotency of stem cells	27	0.01	BMI1, FGFR1, HRAS, APC2, GRB2, IL6ST, SOX2, BMPR2, CTNNB1, ACVR1C, KRAS, WNT6, PIK3R1, PIK3R2, APC, AKT2, DVL3, TBX3, MAP2K2, OTX1, SMAD4, LIFR, STAT3, DVL1, RIF1, MAPK12, BMPR1A
mTOR signaling pathway	14	0.01	CAB39L, STK11, RPS6KB2, IRS1, RRAGB, EIF4B, AKT1S1, EIF4E, ULK3, MLST8, PRKAA2, PIK3R1, AKT2, PIK3R2
Wnt signaling pathway	26	0.02	APC2, PPP3R1, PPP3R2, CTNNB1, PLCB3, PRKACB, SOX17, WNT6, NFATC2, NFATC3, FOSL1, APC, CSNK1A1, TBL1XR1, DVL3, CTBP1, ROCK2, SMAD4, DVL1, CCND1, CCND3, PSEN1, CSNK1E, CCND2, SFRP2, SIAH1
Rap1 signaling pathway	36	0.02	FGFR1, TLN1, HRAS, GNAI1, ADORA2A, PGF, FGF14, EFNA1, EFNA3, CTNND1, ITGAM, CTNNB1, PLCB3, PFN2, KRAS, RAPGEF4, RAPGEF2, RAPGEF1, INSR, PIK3R1, PIK3R2, AKT2, ACTB, PARD6B, MAP2K2, GRIN1, MET, SIPA1L3, RGS14, DOCK4, VEGFB, PRKD2, MAPK12, KRIT1, PARD6G, CRK
TNF signaling pathway	21	0.02	CEBPB, CREB3, PTGS2, CXCL3, CXCL2, FADD, CREB5, JUNB, VCAM1, CASP10, FOS, TNFRSF1B, ATF4, RPS6KA4, MAPK12, PGAM5, CASP8, TNFAIP3, PIK3R1, AKT2, PIK3R2
Cell cycle	23	0.03	ANAPC2, FZR1, YWHAZ, E2F4, E2F5, DBF4, SMAD4, TTK, PRKDC, WEE1, CDK2, TGFB2, CCND1, CDKN1A, YWHAH, CDKN1B, CCND3, CCND2, MDM2, ANAPC7, GADD45B, GADD45A, STAG2

**Table 4:** Top 10 statistically relevant pathways derived from the common 2027-gene signature of U87 glioblastoma cells exposed to 2 and 10 Gy of proton irradiation under hypoxia condition.

Pathways	Gene Count	*p* Value	Genes
Hippo signaling pathway	33	2.22 × 10^9^	YWHAZ, SOX2, BMPR2, LATS1, CTNNB1, TGFB2, WNT3, SERPINE1, DLG4, YAP1, WNT6, PPP2R2C, APC, ACTB, DVL3, PARD6B, NF2, SMAD4, PPP1CC, PPP1CB, TP73, CTNNA2, DVL1, CCND1, YWHAH, RASSF6, CCND3, CSNK1E, CCND2, BBC3, RASSF1, PARD6G, BMP8B
Proteoglycans in cancer	37	2.64 × 10^11^	CAV2, LUM, PPP1R12C, SDC4, MMP2, PDCD4, PXN, IQGAP1, CTNNB1, TGFB2, KRAS, WNT3, ANK2, GAB1, PPP1R12A, MSN, PRKACB, PIK3R3, WNT6, PIK3R1, TWIST1, PIK3R2, AKT2, ACTB, MAP2K2, ROCK2, MET, ITGA2, FLNC, ARHGEF12, PPP1CC, PPP1CB, STAT3, FLNA, CCND1, MAPK12, HBEGF
Endocytosis	40	1.42 × 10^12^	FGFR2, CAV2, CHMP3, WASH1, CAPZA2, STAM2, ASAP2, PIP5K1B, PML, ASAP1, HSPA1A, ARF6, PIP5K1A, AMPH, CHMP2B, SH3GLB2, RAB11B, DNAJC6, RAB11A, NEDD4L, AGAP3, EHD4, GIT1, PARD6B, VTA1, HLA-A, HLA-C, HLA-B, HLA-E, RAB11FIP4, EPS15, ACAP3, AP2A1, ACAP2, RAB5A, SMURF2, PARD6G, PDCD6IP, ARAP2, SH3GL1
p53 signaling pathway	17	1.85 × 10^12^	ZMAT3, RRM2B, CCNG2, SESN1, CDK2, TP73, CCND1, CCND3, BBC3, CCND2, SERPINB5, RRM2, BAX, CASP8, SERPINE1, SIAH1, GADD45B
Focal adhesion	34	5.50 × 10^11^	CAV2, TLN1, XIAP, TLN2, PGF, PPP1R12C, ARHGAP35, PXN, CTNNB1, MYL9, PPP1R12A, COL6A2, SHC1, PIK3R3, RAPGEF1, PIK3R1, PIK3R2, AKT2, ACTB, ROCK2, MYLK3, MET, ITGA2, FLNC, PPP1CC, PPP1CB, COL4A6, FLNA, CCND1, CCND3, ITGA6, CCND2, COL24A1, CRK
Signaling pathways regulating pluripotency of stem cells	25	0.001	FGFR2, BMI1, IL6ST, SOX2, BMPR2, CTNNB1, ACVR1C, WNT3, KRAS, WNT6, PIK3R3, PIK3R1, PIK3R2, APC, AKT2, DVL3, MAP2K2, OTX1, SMAD4, LIFR, STAT3, DVL1, RIF1, MAPK12, JAK3
FoxO signaling pathway	24	0.001	STK11, MAP2K2, PRKAG2, SMAD4, CCNG2, IRS1, CDK2, STAT3, SOD2, TGFB2, CCND1, PLK4, KRAS, CDKN1B, MAPK12, CSNK1E, CCND2, PRKAA2, PIK3R3, GADD45B, INSR, PIK3R1, AKT2, PIK3R2
Wnt signaling pathway	23	0.004	CSNK1A1, DVL3, TBL1XR1, CTBP1, ROCK2, SMAD4, PPP3R1, DKK4, DVL1, CTNNB1, CCND1, WNT3, SOST, CCND3, CSNK1E, SFRP2, CCND2, SIAH1, PRKACB, WNT6, NFATC2, FOSL1, APC
Rap1 signaling pathway	31	0.005	FGFR2, FGF5, TLN1, TLN2, PGF, GNAI1, EFNA3, CTNNB1, PFN2, KRAS, GRIN2B, RAPGEF4, RAPGEF2, PIK3R3, INSR, RAPGEF1, PIK3R1, PIK3R2, AKT2, ACTB, FYB, PARD6B, MAGI1, MAP2K2, MET, SIPA1L3, DOCK4, MAPK12, KRIT1, PARD6G, CRK
VEGF signaling pathway	11	0.04	KRAS, MAPK12, PTGS2, MAP2K2, PPP3R1, NFATC2, PIK3R3, PIK3R1, PXN, AKT2, PIK3R2

**Table 5:** Pubmatrix analysis of the top 10 statistically relevant pathways (*p* values < 0.05) obtained from the 2898-gene signature, upregulated by the combined treatment hypoxia/PT with 2 Gy. Glioblastoma Multiforme (GBM); radiotherapy (RT); proton therapy (PT); ionizing radiation (IR).

PubMatrix	Hypoxia	GBM	RT	PT	Cancer	IR	Cell Death	Cell Cycle	Hif1-Alpha
Ubiquitin-mediated proteolysis	85	10	11	0	1043	54	346	802	1
Regulation of actin cytoskeleton	106	65	12	3	2466	32	610	1180	1
Rap1 signaling pathway	10	5	3	0	322	3	74	92	1
Protein processing in endoplasmic reticulum	184	27	17	4	1676	27	1640	506	0
Proteoglycans in cancer	195	284	339	11		81	1158	1299	4
Platelet activation	584	136	151	101	5387	268	1651	1857	2
Ras signaling pathway	266	200	201	13	11,296	201	2907	3446	7
Glutamatergic synapse	48	3	1	1	95	1	144	50	0
Wnt signaling pathway	310	209	191	11	10,182	70	2563	2294	4
FoxO signaling pathway	53	8	3	0	491	9	329	265	5

**Table 6:** Pubmatrix analysis of the top 10 statistically relevant pathways (*p* values < 0.05) obtained from the 3740-gene signature upregulated by the combined treatment hypoxia/PT with 10Gy. Glioblastoma Multiforme (GBM); radiotherapy (RT); proton therapy (PT); ionizing radiation (IR).

PubMatrix	Hypoxia	GBM	RT	PT	Cancer	IR	Cell Death	Cell Cycle	Hif1-Alpha
PI3K-Akt signaling pathway	1063	427	368	8	12,427	191	6798	3323	26
Insulin signaling pathway	591	89	82	8	6338	100	3505	2647	16
Platelet activation	584	136	151	101	5387	268	1651	1857	2
Endocytosis	384	245	274	107	13,851	391	6505	3903	2
Wnt signaling pathway	310	209	191	11	10,182	70	2563	2294	4
Ras signaling pathway	266	200	201	13	11,296	201	2907	3446	7
Focal adhesion	261	255	127	3	7394	91	1858	1874	3
cAMP signaling pathway	248	47	29	4	2908	117	1273	1490	6
Proteoglycans in cancer	195	284	339	11	13,155	81	1158	1299	4
Protein processing in endoplasmic reticulum	184	27	17	4	1676	27	1640	506	0

## Data Availability

Not applicable.
